# Overweight trajectory and cardio metabolic risk factors in young adults

**DOI:** 10.1186/s12887-019-1445-3

**Published:** 2019-03-11

**Authors:** Gabriela Callo Quinte, Fernando Barros, Denise Petrucci Gigante, Isabel Oliveira de Oliveira, Janaína Vieira dos Santos Motta, Bernardo Lessa Horta

**Affiliations:** 1 Postgraduate Program in Epidemiology, Federal University of Pelotas, Caixa postal 354, Marechal Deodoro, 1160, Pelotas, RS 96020-220 Brazil; 2 Postgraduate Program in Health and Behaviour, Catholic University of Pelotas, Pelotas, Brazil

**Keywords:** Obesity, Trajectory, Cardiometabolic risk factors, Young adults

## Abstract

**Background:**

Obesity is one of the conditions that increases the risk of cardiovascular disease. Studies about obesity trajectory and cardio metabolic outcomes at adulthood are still scarce. Therefore, we aimed to assess the association between patterns of overweight over the life-course and cardio metabolic risk factors in young adults.

**Methods:**

In 1982, the maternity hospitals in Pelotas were visited daily and those newborns whose family lived in the urban area of the city were identified (*n* = 5914), and have prospectively followed for several occasions. Weight and height were measured at every visit. BMI-for-age z-score was calculated using the WHO Child Growth Standards. Overweight and obesity were defined as a BMI greater than or equal to 25 kg/m2 and 30 kg/m2 respectively. This was the definition adopted for evaluations overweight and obesity at 30 years. The participants were divided into eight groups according to the presence of overweight or obesity in childhood, adolescence and adulthood. Blood pressure, random blood glucose, HDL cholesterol, LDL cholesterol triglycerides and fat mass were measured.

**Results:**

From 2219 participants with anthropometric data in childhood, adolescence and adulthood, 25% never had been overweight, whereas 11.6% were overweight in the three periods. Random blood glucose, SBP and DBP were higher among those subjects who were always overweight/ obese or only overweight/obese during adolescence and adulthood. The participants who were never overweight/obese or only in childhood or adolescence had a lower cardiovascular risk profile (higher HDL cholesterol, lower blood pressure, lower random glucose, lower LDL cholesterol) at 30 years. Fat mass captured from 25 to 100% of the association of overweight and obesity trajectory with cardiometabolic risk factors.

**Conclusions:**

The tracking of overweight/obesity is associated with an adverse cardio metabolic profile and this association is largely mediated by fat mass in adulthood.

**Electronic supplementary material:**

The online version of this article (10.1186/s12887-019-1445-3) contains supplementary material, which is available to authorized users.

## Background

Cardiovascular diseases (CVD) remain the major cause of heath loss and accounting for 17.5 million deaths (31% of all deaths) worldwide [1, 2]. One of the conditions that increases the risk of cardiovascular death is obesity [3]. Obese subjects are almost twice as likely to develop CVD [4]. It has been estimated that 35.8 million (2.3%) of Disability Adjusted Life Years (DALYs) around the world are due to overweight or obesity and every year at least 2.8 million people die as a result of being overweight or obese [5]. Because the prevalence of obesity has increased, its contribution to the burden of disease must also have risen [6].

There is evidence that childhood obesity is considered as a cardio metabolic risk predictor in adulthood [7]. The association between obesity and cardiovascular disease would be mainly mediated by increased level of metabolic cardiovascular risk factors [8], since obesity is associated with higher blood pressure [9], Low Density Lipoprotein (LDL) cholesterol, triglycerides, blood glucose and lower High Density lipoprotein (HDL) cholesterol [7, 8, 10–13].

A systematic review has shown that excessive weight gain during the life course increases the risk of hypertension [14]. Another review showed that the tracking of childhood weight into adulthood is moderated for overweight and obese youth [15]. Most of these studies considered only two time points and were conducted in high-income countries.

Because of literature about obesity trajectory and cardio metabolic outcomes at adulthood is still scarce, this study aimed at assessing the association between patterns of overweight over the life-course and cardio metabolic risk factors (random glucose, blood pressure, LDL cholesterol, HDL cholesterol and triglycerides) at 30 years of age.

## Methods

In 1982, we identified all hospital births in Pelotas, a Southern Brazilian city. The liveborns whose families lived in the urban area were examined and their mothers interviewed (*n* = 5914). These individuals were followed up on various occasions. In childhood (2/4 yrs), the subjects were weighed and measured using portable scales and stadiometers by trained staff [16]. During adolescence, in 2000, male subjects were identified while they were participating in the mandatory military recruitment (18 yrs) and were interviewed during the military medical examination. In 2001, a systematic sample of 70 census tracts was selected and a census was carried out in these tracts in search of cohort members, who were interviewed and examined [17]. In 2012–13 (mean age of 30.2 years), the cohort members were contacted and invited to visit the research clinic for interview, examination, and donation of a blood sample [18]. Weight was measured using the Bod POD® scale and height with a portable stadiometer (aluminum and wood) with accuracy of 100 g and 0.1 cm, respectively.

Body mass index (BMI) was estimated from weight and height measurements. Overweight and obesity in childhood (2 and/or 4 years) and adolescence (19 and 20 years), were defined according to specific cutoff points for sex and age (BMI/age), as proposed by World Health Organization (WHO) [19]. Overweight and obesity were defined as a BMI greater than or equal to 25 kg/m2 and 30 kg/m2 respectively [2, 20]. This was the definition adopted for evaluations overweight and obesity at 30 years. Based on these definitions, the participants were categorized into eight groups according to the presence of overweight or obesity in childhood, adolescence and adulthood. i.e.: never obese, childhood only, adolescence only, adulthood only, childhood and adolescence, childhood and adulthood, adolescence and adulthood and always. For the analysis, we collapsed the eight groups into six putting together childhood only, adolescent only and childhood and adolescent only. Thus, the new six groups were: never, childhood/adolescence only, adulthood only, childhood and adulthood, adolescence and adulthood and always.

In the present manuscript, we evaluated the association of the overweight/obesity trajectory with the following metabolic cardiovascular risk factors at age 30 years:

- Blood pressure was measured using a wrist digital sphygmomanometer (Omron HEM-742) at the beginning and at the end of the interview with the participant seated and the arm supported at chest level, and the mean of the two measurements was used in the present analyses.
- Random blood glucose was measured using an automatic enzymatic colorimetric method in chemistry analyzer BS-380, Mindray (Shenzhen Mindray Bio-Medical Electronics Co., Ltd., China). Because glucose levels vary according to fasting time, estimates were adjusted for time since last meal using linear regression models with glucose as the dependent variable, and time since the last meal as independent variable.
- HDL cholesterol, LDL cholesterol and triglycerides were measured enzymatically (Shenzhen Mindray Bio-Medical Electronics Co., Ltd., China).

Fat mass was estimated by air displacement plethysmography (BOD POD), using the Siri equation [21].

The following variables were considered as confounders: family income at birth (in minimum wages), maternal schooling at birth (in completed years), maternal smoking during pregnancy (yes/no), skin color, sex, physical activity at 30 years, assessed using the International Physical Activity Questionnaire (IPAQ) [22] large version, income at 30 years and fasting time (used for random glucose, HDL cholesterol, ldl cholesterol and triglycerides). In the crude analysis, analysis of variance was used to compare the means. In the multivariate analysis, we used multiple linear regression to obtain estimates that were adjusted for confounding. We used G computation to assess the mediating effect of fat mass at 30 years in the association between overweight trajectory and cardio metabolic risk factors and estimated the natural direct effect (NDE) and natural indirect effects (NIE). The NDE represents the effect that goes directly to the outcome from the exposure without passing through the mediator (fat mass). The NIE is the effect that passes through the mediator (fat mass) [23]. In the G-computation analysis, base confounders included the variables considered as confounders in the linear regression. Physical activity at 30 years and current income were included as post confounders (Additional file 1: Figure S1), i.e., variables that could also confound the relationship between the mediator (fat mass) and the outcomes (cardio metabolic risk factors).

Study protocols were approved by the Ethics Committee of the Federal University of Pelotas. Verbal consent was obtained from those responsible for the children in the initial phases of the study (1982–1986). All participants signed an informed consent form before any interviews and evaluations.

## Results

**Fig. 1:**
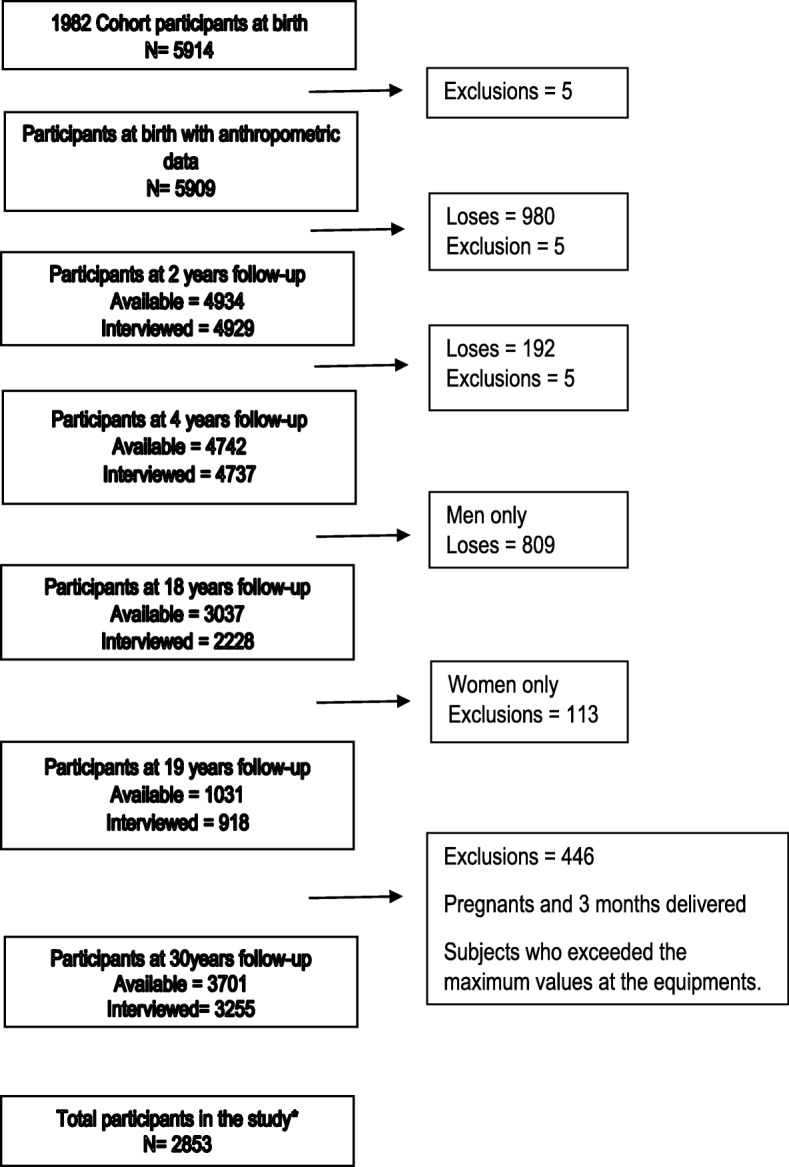
Flow chart of the Pelotas 82 cohort Participants. *All the subjects that had height and weight measurement during childhood, adolescence and adulthood

In 2012–13, 3701 subjects were interviewed, which added to the 325 known to have died, represented a follow-up rate of 68.1%. In the present study, we analyzed 2219 subjects who had weight and height measurement in childhood, adolescence and adulthood (Fig. 1).

About one of every four subjects were never overweight, whereas 11.6% were overweight in the three periods. Table 1 shows the socio-demographic characteristics of study participants, most of them were white (75.5%), their mothers had completed 5 to 8 school years (43.1%) and 6.2% were low birth weight.

**Table 1:** Characteristics of Pelotas Cohort participants who had weight and height data in childhood, adolescence and adulthood

Variable	N	% or Mean (SD)
Sex		
Male	2002	(70,2)
Female	851	(8, 29)
Ethnicity		
White	1721	(75,5)
Black/brown	486	(3, 21)
Others	74	(2, 3)
Family income at birth (tertile)		
1(poorest)	717	(33,3)
2	786	(33,4)
3	716	(33,3)
Mother schooling at birth (years)		
0–4	928	(32.6)
5–8	1229	(43.1)
9–11	311	(10.9)
12 or more	382	(13.4)
Birth weight (g)		
< 2500	178	(2, 6)
2500 a 3499	1723	(60,4)
≥ 3500	951	(33,4)
Gestational age (weeks)		
< 37	119	(5.2)
37–41	1931	(84.5)
≥42)	234	(10.3)
Childhood	684	(4, 25)
Overweight	216	(8.0)
Obesity		
Adolescence	234	(11.7)
Overweight	102	(5.1)
Obesity		
Adulthood		
Overweight	836	(37.8)
Obese	509	(23.0)
Cardio metabolic risk factors at 30 years		
Systolic blood pressure (mmHg)		123.8(13.9)
Diastolic blood pressure (mmHg)		75.9(9.5)
Random glucose (mg/dl)		90.0(25.2)
HDL cholesterol (mg/dl)		56.4(13.2)
LDL cholesterol (mg/dl)		110.7(28.9)
Triglycerides (mg/dl)		128.0(111.7)
Fat mass (%)		28.5(10. 8)
Overweight patterns		
Never	762	(26.7)
Chilhood or adolescence only	544	(19.1)
Adulthood only	553	(19.4)
Childhood + adulthood	530	(18.5)
Adolescence + adulthood	134	(4.7)
Always	330	(11.6)
Total	2853	

Table 1 The association of these variables with the exposure and outcomes is provided in Additional file 2: Table S1, Additional file 3: Table S2.

Tables 2 and 3 show that systolic and diastolic blood pressure were higher among those subjects who were always overweight/obese or during adolescence and adulthood. On the other hand, those who were overweight/obese only in childhood or adolescence had similar crude mean values to those who were never considered as overweight. For random blood glucose, we observed a similar pattern of association. These associations were observed even after controlling for possible confounding variables.

**Table 2:** Associations between overweight during childhood, adolescence and adulthood and cardio metabolic outcomes in the 82 Pelotas cohort

		SBP (mmHg)		DBP (mmHg)		Random glucose (mg/dl)		HDL Cholesterol (mg/dl)		LDL cholesterol (mg/dl)		Triglycerides (mg / dl)	
	n	Mean (95%CI)	β* (95%CI)	Mean (95%CI)	β* (95%CI)	Mean (95%CI)	β** (95%CI)	Mean (95%CI)	β** (95%CI)	Mean (95%CI)	β** (95%CI)	Mean (95%CI)	β** (95%CI)
	2853												
Overweight pattern													
Never	762	119.4 (118.4;120.4)	Reference	72.9 (72.2;73.6)	Reference	86.2 (84.7;87.8)	Reference	60.1 (58.9;61.3)	Reference	104.7 (102.3;107.0)	Reference	101.7 (96.6;106.9)	Reference
Chilhood or adolescence only^a^	544	119.9 (118.5;121.3)	0.4 (− 1.2; 2.0)	72.4 (71.5;73.3)	− 0.6 (− 1.8;0.7)	84.8 (83.3;86.2)	− 2.1 (− 5.5;1.3)	59.7 (58.3;61.1)	− 0.6 (− 2.3; 1.1)	100.8 (97.8;103.7)	− 4.2 (− 8.1;-0.3)	91.5 (84.8;98.2)	0.9 (0.8;1.0)
Adulthood only^b^	553	125.1 (124.0:126.3)	4.8 (3.3;6.2)	77.1 (73.3;77.8)	3.9 (2.8;5.0)	90.3 (88.8;91.7)	3.2 (0.1;6.2)	54.1 (53.1;55.2)	−5.3 (−6.9;-3.8)	115.9 (113.5;118.4)	11.0 (7.6;14.5)	142.9 (134.6;151.1)	1.3 (1.3;1.4)
Childhood + adulthood	530	125.3 (124.1;126.5)	4.1 (2.6;5.6)	76.7 (75.9;77.5)	3.4 (2.3;4.5)	91.3 (89.3;93.4)	4.1 (1.0;7.2)	54.4 (53.2;55.5)	−4.8 (−6.4;-3.3)	114.5 (111.8;117.1)	9.0 (5.4;12.5)	138.6 (129.6;147.5)	1.3 (1.2;1.4)
Adolescence + adulthood	134	128.9 (126.3;131.5)	9.6 (7.2;12.1)	81.3 (79.4;83.3)	8.5 (6.6;10.4)	98.5 (89.2;107.7)	12.5 (7.1;17.7)	55.6 (53.0;58.2)	−4.8 (−7.3;-2.2)	115.7 (109.6;121.9)	10.9 (4.9;16.8)	172.9 (114.7;231.1)	1.4 (1.3;1.6)
Always	330	129.9 (128.0;131.9)	9.8 (8.0;11.3)	80.6 (79.3;81.8)	7.5 (6.2;8.8)	97.8 (92.4;103.1)	10.8 (7.0;14.5)	53.0 (51.5;54.5)	−7.0 (−8.8;-5.1)	115.6 (112.1;119.0)	10.1 (5.8;14.3)	160.0 (145.1;175.0)	1.5 (1.3;1.6)

*Adjusted for sex, birth weight, skin color, family income at birth, maternal schooling and maternal smoking during pregnancy

**Adjusted for sex, birth weight, skin color, family income at birth, maternal schooling, maternal smoking during pregnancy and fasting time

^a^Overweight/ obesity (BMI/age) WHO charts, 2006

^b^Adults only: overweight BMI ≥25; obesity BMI ≥ 30

**Table 3:** Associations between obesity during childhood, adolescence and adulthood and cardio metabolic outcomes in the 82 Pelotas cohort

		SBP (mmHg)		DBP (mmHg)		Random glucose (mg/dl)		HDL Cholesterol (mg/dl)		LDL cholesterol (mg/dl)		Triglycerides (mg / dl)	
	n	Mean (95%CI)	β* (95%CI)	Mean (95%CI)	β* (95%CI)	Mean (95%CI)	β** (95%CI)	Mean (95%CI)	β** (95%CI)	Mean (95%CI)	β** (95%CI)	Mean (95%CI)	β** (95%CI)
	2853												
Obesity pattern													
Never	2036	121.6 (121.0;122.3)	Reference	74.1 (73.7;74.6)	Reference	87.6 (86.7;88.6)	Reference	57.5 (58.9;58.2)	Reference	108.4 (107.0;109.9)	Reference	115.9 (111.8;120.0)	Reference
Chilhood or adolescence only^a^	253	122.4 (120.6;124.2)	−0.5 (− 2.2;1.3)	74.2 (72.9;75.5)	− 0.2 (− 1.6;1.1)	88.3 (85.6;90.1)	0.1 (− 3.6;3.9)	57.0 (55.1;58.9)	−0.3 (− 2.2; 1.6)	108.8 (104.7;113.0)	− 0.3 (− 4.74.1)	112.9 (99.0;126.9)	1.0 (0.9;1.1)
Adulthood only^b^	343	129.8 (128.3;131.2)	8.4 (7.0;9.8)	81.5 (80.5;82.5)	7.4 (6.4;8.5)	94.6 (91.2;98.1)	7.2 (4.2;10.1)	53.1 (51.8;54.4)	−4.5 (−6.0;-3.0)	117.9 (114.8;121.0)	9.4 (5.9;12.8)	169.1 (148.6;189.6)	1.4 (1.3;1.5)
Childhood + adulthood	85	127.0 (124.7;1291.3)	4.6 (1.9;7.3)	79.8 (78.1;81.5)	5.5 (3.5;7.5)	88.8 (85.6;92.0)	0.6 (−5.1;6.3)	52.4 (50.0;54.8)	−4.8 (−7.7;-2.0)	119.3 (112.4;126.1)	10.1 (3.4;16.8)	156.5 133.2;179.8	1.3 (1.2;1.5)
Adolescence + adulthood	85	134.3 (130.2;138.3)	13.3 (10.6;16.0)	83.8 (81.4;86.2)	9.9 (7.8;11.9)	114.5 (101.0;128.0)	27.2 (21.4;32.9)	52.5 (49.6;55.4)	−5.6 (−8.5;-2.7)	118.3 (110.9;125.8)	9.7 (2.9;16.5)	172.8 (142.7;202.9)	1.5 (1.3;1.7)
Always	51	137.9 (131.6;144.2)	14.8 (10.6;18.0)	85.4 (81.8;89.0)	10.9 (8.1;13.7)	108.0 (87.9;128.1)	19.2 (11.4;26.9)	51.5 (47.1;55.9)	−5.4 (−9.4;-1.5)	112.2 (102.8;121.7)	1.9 (−7.2;11.1)	184.4 (132.5;236.3)	1.4 (1.2;1.6)

*Adjusted for sex, birth weight, skin color, family income at birth, maternal schooling and maternal smoking during pregnancy

**Adjusted for sex, birth weight, skin color, family income at birth, maternal schooling, maternal smoking during pregnancy and fasting time

^a^Overweight/ obesity (BMI/age) WHO charts, 2006

^b^Adults only: overweight BMI ≥25; obesity BMI ≥ 30

Concerning triglycerides, HDL and LDL cholesterol, the participants who were never overweight/obese or only in childhood or adolescence had a lower cardiovascular risk profile (higher HDL, lower LDL and triglycerides), whereas those who were overweight/obese at adulthood (independent of episodes of overweight/obesity at any other period) had the lowest levels of HDL and highest LDL and triglycerides. The direction of the associations was similar for men and women (data not shown). However, we found that after controlling adult BMI, the trajectory association decreased (Additional file 4: Table S3).

In the mediation analysis, we observed that fat mass captured from 25 to 100% of the effect of overweight and obesity trajectory on the cardio metabolic risk factors (Additional file 5:Table S4, Additional file 6:Table S5).

## Discussion

In a population that has been prospectively followed since birth, we observed that overweight throughout life course or during adolescence and adulthood was associated with higher systolic and diastolic blood pressure, random glucose, and lower HDL cholesterol, whereas being overweight only in adulthood showed an intermediate effect. On the other hand, LDL cholesterol and triglycerides were associated with current body mass index, independent of what had been observed at earlier ages. After controlling adult BMI, the trajectory association decreased reinforcing our thesis that what matters is the current BMI. In addition, we observed that a great part of these associations was explained by fat mass in adulthood.

We found that individuals exposed to overweight in childhood and/or adolescence and become non obese in adulthood showed similar cardio metabolic profile to those who had never presented overweight. These findings are in agreement with Juonala et al., who observed that a decrease in adiposity from childhood to adulthood was associated with a marked reduction in the risk of hypertension, type 2 diabetes, and dyslipidemia [10]. Furthermore, a pooled analysis from three British birth cohorts reported an increased risk of type 2 diabetes, when overweight presented at different stages of life (childhood, adolescence and adulthood), but overweight in childhood was not associated with higher risk for subjects who were not overweight in adulthood [24]. In addition, a recent study using observational data suggest that stabilizing BMI among obese adults could help limit their adverse CVD risk profiles and that reversing high BMI in young adulthood may lead to better cardio metabolic profiles than remaining stable overweight [25].

A previous study from this same cohort showed that a continuous exposure to overweight was associated with higher fat mass in adulthood showing a cumulative effect, whereas those who were overweight only at childhood or adolescence presented similar body composition to those who were never overweight [26]. In this study, most of the effect of overweight trajectory on the cardio metabolic risk factors was captured by fat mass. Thus, because fat mass is positively associated with cardio metabolic risk [27–29], it is not surprising that fat mass is the main mediator of the association between overweight tracking and cardio metabolic risk. Indeed, recovering normal weight at adulthood reduce fat mass and then, the cardio metabolic risk [10].

We were unable to track down 31.9% of the cohort members. However, the follow-up rate was slightly higher among those in the intermediate socioeconomic groups and reasonably similar among different subgroups (ranging from 60 to 75% in all variables studied) [18]. Therefore, it is unlikely that the observed association was due to attrition bias. Blood samples were obtained at random and only 7.2% of the subjects reported a fasting time of 12 h of more. On the other hand, fasting time was not associated with episodes of overweight/obesity over the life course. Therefore, it is unlikely that the observed associations were due to a differential misclassification. Indeed, we would expect a non-differential misclassification that would tend to underestimate the magnitude of the associations. In spite of BMI has some limitations [30], it is still the most used worldwide index to estimate overweight/obesity. We acknowledge the relevance of also examining the consequences of small changes in BMI. Nonetheless, we were willing to assess the effect of changing overweight/obesity status through the life cycle. Our model points out the potential benefits of early intervention to reduce overweight and minimize individuals’ exposure to cardio metabolic risk factors. In terms of public health, this analysis brings a clearer message to policy makers.

## Conclusions

In summary, our results indicate that the tracking of overweight/obesity is associated with an adverse cardio metabolic profile and this association is largely mediated by fat mass in adulthood. However, interruption of tracking can reverse this effect. This evidence highlights the importance of not only early intervention to reduce obesity and therefore, cardio metabolic risk factors, but also during the entire life course.

## Additional files

Additional file 1: **FigureS1**. Direct Acyclic Graph of the association between overweight trajectory and cardio metabolic risk factors (i.e. systolic blood pressure). BC = Base confounders, which included, birth weight, mother’s schooling, smoking and income at birth. PC=Post Confounders, which included, income at 30 years and physical activity at 30 years. (DOCX 20 kb)

Additional file 2: **Table S1**. Overweight pattern according to confounding variables. (DOCX 14 kb)

Additional file 3: **Table S2**. Cardio metabolic risk factors, according to confounding variables. (DOCX 15 kb)

Additional file 4: **Table S3**. Associations between overweight/obesity during childhood, adolescence and adulthood and cardiovascular outcomes in the 82 Pelotas cohort adjusted for BMI at 30 years. * Adjusted for BMI at 30 years, sex, birth weight, skin color, family income at birth, maternal schooling, maternal smoking during pregnancy. ** Adjusted for BMI at 30 years, sex, birth weight, skin color, family income at birth, maternal schooling, maternal smoking during pregnancy and fasting time (DOCX 15 kb)

Additional file 5: **Table S4**: Mediation analysis of the association between overweight trajectory and cardio metabolic risk factors. Mediated by fat mass. *Adjusted for base confounder: low birth weight, skin color, mother schooling, sex, maternal smoking in pregnancy, and family income at birth and post confounders: physical activity at 30 years. CI=Confidence Interval. (DOCX 12 kb)

Additional file 6: **Table S5**: Mediation analysis of the association between obesity trajectory and cardio metabolic risk factors. Mediated by fat mass. *Adjusted for base confounder: low birth weight, skin color, mother schooling, sex, maternal smoking in pregnancy, and family income at birth and post confounders: physical activity at 30 years. CI=Confidence Interval. (DOCX 12 kb)

## Abbreviations

BMI: Body Mass Index
CVD: Cardiovascular diseases
DALY’s: Disability Adjusted Life Years
HDL: High Density lipoprotein
IPAQ: International Physical Activity Questionnaire
LDL: Low Density Lipoprotein
NDE: Natural direct effect
NIE: natural indirect effect
WHO: World Health Organization
