# Supplementary material for: Overweight trajectory and cardio metabolic risk factors in young adults
Source: BMC Pediatr. 2019 Mar 11;19:75. doi: 10.1186/s12887-019-1445-3 (PMC6410517; doi:10.1186/s12887-019-1445-3)
Supplement: Supplementary file 1 — FigureS1. Direct Acyclic Graph of the association between overweight trajectory and cardio metabolic risk factors (i.e. systolic blood pressure). BC = Base confounders, which included, birth weight, mother’s schooling, smoking and income at birth. PC=Post Confounders, which included, income at 30 years and physical activity at 30 years. (DOCX 20 kb) [file 12887_2019_1445_MOESM1_ESM.docx]

BC Fat mass PC

Overweight trajectory Systolic blood pressure
