# Supplementary material for: Overweight trajectory and cardio metabolic risk factors in young adults
Source: BMC Pediatr. 2019 Mar 11;19:75. doi: 10.1186/s12887-019-1445-3 (PMC6410517; doi:10.1186/s12887-019-1445-3)
Supplement: Supplementary file 2 — Table S1. Overweight pattern according to confounding variables. (DOCX 14 kb) [file 12887_2019_1445_MOESM2_ESM.docx]

|  | Never overweight | Childhood or adolescence only | Adulthood only | Childhood + adulthood | Adolescence + adulthood | Always |  |
| --- | --- | --- | --- | --- | --- | --- | --- |
| Family income at birth(tertiles) | p=0.031 |  |  |  |  |  |  |
| 1 | 262 (28.8) | 168 (18.4) | 171 (18.8) | 175 (19.2) | 53 (5.8) | 82 (9.0) | 911 |
| 2 | 263 (25.9) | 187 (18.5) | 217 (21.4) | 185 (18.3) | 39 (3.9) | 122 (12.0) | 1013 |
| 3 | 237 (25.5) | 189 (20.3) | 165 (17.8) | 170 (18.3) | 42 (4.5) | 126 (13.6) | 929 |
| Sex | p<0.05 |  |  |  |  |  |  |
| Male | 508 (25.4) | 370 (18.5) | 396 (19.8) | 410 (20.5) | 83 (4.2) | 235 (11.7) | 2002 |
| Female | 254 (29.9) | 174 (20.5) | 157 (18.5) | 120 (14.1) | 51 (6.0) | 95 (11.2) | 851 |
| Skin color | p=0.268 |  |  |  |  |  |  |
| White | 420 (2.4) | 277 (16.1) | 392 (22.8) | 356 (20.7) | 76 (4.4) | 200 (11.6) | 1721 |
| Black | 117 (24.0) | 64 (13.2) | 109 (22.4) | 109 (22.4) | 32 (6.6) | 55 (11.3) | 486 |
| Others | 10 (13.5) | 11 (14.9) | 20 (27.0) | 18 (24.3) | 3 (4.1) | 12 (16.2) | 74 |
| Birth weight (g) | p<0.05 |  |  |  |  |  |  |
| < 2500 | 67(37.6) | 26(14.6) | 44(24.7) | 23(3.9) | 7(3.9) | 11(6.2) | 178 |
| ≥2500 | 695(26.0) | 518(19.4) | 509(19.0) | 506(18.9) | 127(4.8) | 319(11.9) | 2674 |
| Maternal education at delivery(y) | p=0.019 |  |  |  |  |  |  |
| 0 - 4 | 275(29.6) | 160(17.2) | 172(18.5) | 174(18.8) | 57(6.1) | 90(9.7) | 928 |
| 5 - 8 | 310(25.2) | 241(19.6) | 259(21.1) | 227(18.5) | 41(3.3) | 151(12.3) | 1229 |
| 9 - 11 | 84(27.0) | 58(18.7) | 51(16.4) | 59(19.0) | 20(6.4) | 39(12.6) | 311 |
| ≤ 12 | 92(24.1) | 85(22.3) | 69(18.1) | 70(18.3) | 16(4.2) | 50(13.1) | 382 |
| Maternal smoking during pregnacy | p=0.33 |  |  |  |  |  |  |
| Yes | 245(24.7) | 183(18.4) | 201(20.2) | 187(18.8) | 53(5.3) | 124(12.5) | 993 |
| No | 517(27.8) | 361(19.4) | 352(18.9) | 343(18.4) | 81(4.4 | 206(11.1) | 1860 |
| Fasting time | p=0.065 |  |  |  |  |  |  |
| ≤8 | 450(23.9) | 297(15.8) | 444(23.6) | 392(20.8) | 85(8.5) | 215(11.4) | 1883 |
| 8 - 12 | 38(24.7) | 17(11.0) | 36(23.4) | 36(23.4) | 10(6.5) | 17(11.0) | 154 |
| ≥12 | 32(18.8) | 19(11.2) | 32(18.8) | 48(28.2) | 10(5.9) | 29(17.1) | 170 |
