# Supplementary material for: Overweight trajectory and cardio metabolic risk factors in young adults
Source: BMC Pediatr. 2019 Mar 11;19:75. doi: 10.1186/s12887-019-1445-3 (PMC6410517; doi:10.1186/s12887-019-1445-3)
Supplement: Supplementary file 3 — Table S2. Cardio metabolic risk factors, according to confounding variables. (DOCX 15 kb) [file 12887_2019_1445_MOESM3_ESM.docx]

|  | SBP  (mmHg) | DBP  (mmHg) | Random glucose  (mg/dl) | HDL  (mg/dl) | LDL  (mg/dl) | Triglycerides  (mg/dl) |
| --- | --- | --- | --- | --- | --- | --- |
| Family income at birth(tertiles) | p<0.508 | p=0.523 | p=0.461 | p<0.05 | p<0.05 | p<0.05 |
| 1 | 121.2  (120.4-122.1) | 75.2  (74.6-75.8) | 89.7  (88.1-91.2 | 57.6  (56.9-58.4) | 108.5  (106.8-110.3) | 113.9(108.4-119.4) |
| 2 | 121.4  (120.7-122-2) | 75.6  (75.1-76.1) | 90.1  (88.8-91.5) | 57.6  (56.9-58.4) | 110.0  (108.4-111.6) | 123.9(119.0-128.8) |
| 3 | 120.8  (120.0-121.5) | 75.2  (74.7-75.7) | 88.8  (87.3-90.4) | 60.6  (59.8-61.5) | 109.5  (107.8-111.1) | 125.3(118.2-132.3) |
| Sex | p<0.05 | p<0.05 | p<0.05 | p<0.05 | p<0.05 | p<0.05 |
| Male | 128.1  (127.5-128.6) | 77.0  (76.5-77.4) | 92.5  (91.1-93.9) | 53.7  (53.2-54.3) | 112.2  (110.8-113.6) | 140.5(134.5-146.6) |
| Female | 114.5  (114.0-115.1) | 73.9  (73.4-74.3) | 86.7  (85.7-87.6) | 63.4  (62.7-64.0) | 106.6  (105.3-107.9) | 102.4(99.6-105.3) |
| Skin color | p<0.05 | p<0.05 | p=0.359 | p=0.073 | p=0.405 | p<0.05 |
| White | 120.5  (120.0-121.0) | 75.1  (74.7-75.4) | 89.2  (88.3-90.1) | 58.6  (58.1-59.2) | 109.7  (108.6-110.7) | 125.1(121.1-129.1) |
| Black | 123.5  (122.5-124.5) | 76.4  (75.7-77.0) | 90.5  (88.2-92.9) | 59.1  (58.1-60.1) | 108.2  (106.0-110.3) | 107.9(101.1-114.6) |
| Others | 121.8  (119.1-124.4) | 75.3  (73.4-77.1) | 91.3  (86.0-96.7) | 55.9  (53.5-58.3) | 110.8  (104.8-116.8) | 116.7(99.9-133.3) |
| Birth weight (g) | p=0.727 | 0.789 | p=0.153 | p<0.05 | p=0.068 | p=0.695 |
| < 2500 | 121.2  (120.7-121.6) | 75.5  (74.4-76.6) | 89.7  (88.8-90.6) | 58.8  (58.3-59.2) | 109.6  (108.6-110.6) | 118.8(110.0-127.6) |
| ≥2500 | 120.9  (119.1-122.6) | 75.3  (75.0-75.7) | 87.3  (85.5-89.1) | 55.0  (55.4-58.5) | 106.2  (103.0-109.3) | 121.4(117.8-125.0) |
| Maternal schooling at delivery(y) | p=0.129 | p<0.05 | p<0.05 | p<0.05 | p=0.308 | p=0.173 |
| 0 - 4 | 120.8  (120.0-121.6) | 75.0  (74.4-75.5) | 89.0  (87.6-90.4) | 57.4  (56.6-58.2) | 108.5  (106.8-110.2) | 117.0(111.3-122.7) |
| 5 - 8 | 121.5  (120.8-122.2) | 75.8  (75.3-76.2) | 90.8  (89.4-92.2) | 58.0  (57.4-58.7) | 110.3  (108.8-111.7) | 121.4(117.2-125.7) |
| 9 - 11 | 122.1  (120.7-123.4) | 75.8  (74.8-76.7) | 86.6  (85.2-88.1) | 59.7  (58.3-61.1) | 110.0  (107.1-112.9) | 123.7(115.2-132.2) |
| ≤ 12 | 120.2  (119.0-121.4) | 74.7  (73.9-75.5) | 89.2  (86.5-91.9) | 62.6  (61.2-63.9) | 108.0  (105.6-110.5) | 129.0(114.8-143.3) |
| Maternal smoking during pregnancy | p=0.822 | p=0.950 | p<0.05 | p=0.114 | p=0.532 | p=0.447 |
| Yes | 121.2  (120.5-122.0) | 75.3  (74.8-75.9) | 90.9  (89.3-92.5) | 58.1  (57.4-58.8) | 109.1  (108.0-110.3) | 123.0(117.4-128.6) |
| No | 121.1  (120.6-121.7) | 75.4  (75.0-75.7) | 88.8  (87.8-89.8) | 58.9  (58.3-59.5) | 109.8  (108.1-111.4) | 120.2(116.0-124.5) |
| Fasting time | |  | p=0.087 | p=0.171 | p<0.05 | p<0.05 |
| ≤8 |  |  | 89.6  (88.7-90.5) | 58.8  (58.3-59.3) | 108.8  (107.8-109.9) | 123.5(120.2-126.7) |
| 8 - 12 |  |  | 91.6  (87.1-96.1) | 57.5  (55.9-59.1) | 113.6  (109.8-117.5) | 112.4(86.4-138.4) |
| ≥12 |  |  | 86.6  (84.5-88.7) | 57.6  (55.9-59.3) | 111.9  (108.1-115.6) | 102.3(92.9-111.9) |
