# Supplementary material for: Overweight trajectory and cardio metabolic risk factors in young adults
Source: BMC Pediatr. 2019 Mar 11;19:75. doi: 10.1186/s12887-019-1445-3 (PMC6410517; doi:10.1186/s12887-019-1445-3)
Supplement: Supplementary file 4 — Table S3. Associations between overweight/obesity during childhood, adolescence and adulthood and cardiovascular outcomes in the 82 Pelotas cohort adjusted for BMI at 30 years. * Adjusted for BMI at 30 years, sex, birth weight, skin color, family income at birth, maternal schooling, maternal smoking during pregnancy. ** Adjusted for BMI at 30 years, sex, birth weight, skin color, family income at birth, maternal schooling, maternal smoking during pregnancy and fasting time (DOCX 15 kb) [file 12887_2019_1445_MOESM4_ESM.docx]

|  |  | SBP (mmHg) | DBP (mmHg) | Random glucose (mg/dl) | HDL Cholesterol (mg/dl) | LDL cholesterol (mg/dl) | Triglycerides  (mg / dl) |
| --- | --- | --- | --- | --- | --- | --- | --- |
|  |  | β* (95%CI) | β* (95%CI) | β**  (95%CI) | β** (95%CI) | β**  (95%CI) | β**  (95%CI) |
| Overweight pattern | 2853 |  |  |  |  |  |  |
| Never | 762 | Reference | Reference | Reference | Reference | Reference | Reference |
| Chilhood or adolescence only^a^ | 544 | -0.4  (-1.9; 1.2) | -1.3  (-2.5;-0.1) | -2.8  (-6.2;0.6) | -0.2  (-1.9; 1.5) | -5.1  (-9.0;-1.1) | 0.9  (0.8;0.9) |
| Adulthood only^b^ | 553 | -0.9  (-2.6;0.8) | -0.8  (-3.1;0.4) | -2.8  (-6.5;0.8) | -2.7  (-4.5;-0.9) | 4.6  (0.4;8.8) | 1.0  (0.9;1.1) |
| Childhood + adulthood | 530 | -2.0  (-3.8;-0.3) | -1.7  (-3.1;-0.5) | -2.8  (-6.6;0.9) | -1.9  (-3.84;-0.0) | 2.4  (-2.0;6.7) | 1.0  (0.9;1.1) |
| Adolescence + adulthood | 134 | -0.7  (-3.7;2.2) | -.02  (-2.4;2.1) | 1.0  (-5.3;7.4) | 0.3  (-2.9;3.4) | -0.8  (-8.2;6.6) | 0.9  (0.8;1.1) |
| Always | 330 | -1.9  (-4.6;0.6) | -2.4  (-4.4;-0.5) | -2.1  (-7.6;3.5) | -1.3  (-4.1;1.5) | -3.2  (-9.7;3.3) | 0.9  (0.8;1.0) |
|  |  |  |  |  |  |  |  |
|  |  |  |  |  |  |  |  |
| Obesity pattern |  |  |  |  |  |  |  |
| Never | 2036 | Reference | Reference | Reference | Reference | Reference | Reference |
| Chilhood or adolescence only^a^ | 253 | -.3  (-3.2;0.4) | -1.1  (-2.5;0.2) | -1.7  (-5.4;2.3) | 0.8  (-1.2;2.7) | -2.3  (-6.7;2.2 | 0.9  (0.9;1.0) |
| Adulthood only^b^ | 343 | 3.0  (1.1;4.9) | 2.9  (1.4;4.3) | 2.1  (-1.9;6.2) | 0.9  (-1.1;3.0) | -4.9  (-9.6;0.2) | 1.0  (0.9;1.1) |
| Childhood + adulthood | 85 | -1.2  (-4.1;1.8) | 0.6  (-1.6;2.8) | -4.6  (-10.9;6.2) | 0.8  (-2.4;4.0) | -5.1  (-12.5;2.4) | 0.9  (0.8;1.0) |
| Adolescence + adulthood | 85 | 4.8  (1.4;8.2) | 2.7  (0.1;5.2) | 19.3  (12.1;26.5) | 2.7  (-0.9;6.4) | -12.5  (-20.9;-4.1) | 0.8  (0.7;0.9) |
| Always | 51 | 5.4  (1.2;9.6) | 3.4  (0.2;6.5) | 10.7  (1.7;19.6) | 3.4  (-1.2;7.9) | -20.9  (-31.3;-10.4) | 0.7  (0.6;0.9) |
