# Supplementary material for: Overweight trajectory and cardio metabolic risk factors in young adults
Source: BMC Pediatr. 2019 Mar 11;19:75. doi: 10.1186/s12887-019-1445-3 (PMC6410517; doi:10.1186/s12887-019-1445-3)
Supplement: Supplementary file 5 — Table S4: Mediation analysis of the association between overweight trajectory and cardio metabolic risk factors. Mediated by fat mass. *Adjusted for base confounder: low birth weight, skin color, mother schooling, sex, maternal smoking in pregnancy, and family income at birth and post confounders: physical activity at 30 years. CI=Confidence Interval. (DOCX 12 kb) [file 12887_2019_1445_MOESM5_ESM.docx]

| G-Computation estimate (95%CI) | SBP | DBP | Random glucose | HDL Cholesterol | LDL cholesterol | Triglycerides |
| --- | --- | --- | --- | --- | --- | --- |
|  |  |  |  |  |  |  |
| Natural  direct effect | 0.248  (-0.517; 1.014) | -0.449  (-1.036;0.136) | 0.897  (-.864; 2.659) | -.573  (-1.405;.258) | 2.662  (.677; 4.647) | .021  (-.016; .060) |
| Natural indirect effect | 0.915  (0.149; 1.682) | 0.940  (0.349; 1.531) | 0.359  (-1.204; 1.924) | -1.734  (-2.551;-.916) | 4.172  (2.187;6.156) | .093  (.056; .131) |
| Mediated effect (%) | 67 | 100 | 100 | 73 | 59 | 81 |
