# Supplementary material for: Overweight trajectory and cardio metabolic risk factors in young adults
Source: BMC Pediatr. 2019 Mar 11;19:75. doi: 10.1186/s12887-019-1445-3 (PMC6410517; doi:10.1186/s12887-019-1445-3)
Supplement: Supplementary file 6 — Table S5: Mediation analysis of the association between obesity trajectory and cardio metabolic risk factors. Mediated by fat mass. *Adjusted for base confounder: low birth weight, skin color, mother schooling, sex, maternal smoking in pregnancy, and family income at birth and post confounders: physical activity at 30 years. CI=Confidence Interval. (DOCX 12 kb) [file 12887_2019_1445_MOESM6_ESM.docx]

| G-Computation estimate (95%CI) | SBP | DBP | Random glucose | HDL Cholesterol | LDL cholesterol | Triglycerides |
| --- | --- | --- | --- | --- | --- | --- |
|  |  |  |  |  |  |  |
| Natural  direct effect | 0.816  (-.179; 1.81) | 0.887  (0.173; 1.602) | -2.375  (-4.117; -0.633) | -1.720  (-2.722; -.719) | 1.379  (-.974; 3.732) | .034  (-.010; .080) |
| Natural indirect effect | 1.810  (1.037; 2.583) | 1.282  (0.696; 1.867) | 1.431  (-0.119; 2.981) | -.915  (-1.738;-.093) | 4.983  (3.057; 6.910) | .122  (.085; .159) |
| Mediated effect (%) | 55 | 73 | 25 | 31 | 76 | 65 |
